# The geriatric asthma: pitfalls and challenges

**DOI:** 10.1186/s40733-015-0018-y

**Published:** 2016-01-04

**Authors:** Alida Benfante, Nicola Scichilone

**Affiliations:** 1 Dipartimento Biomedico di Medicina Interna e Specialistica, University of Palermo, Istituto Euro-Mediterraneo di Scienza e Tecnologia, via Trabucco 180, 90146 Palermo, Italy; 2 Euro-Mediterranean Institute of Science and Technology, Palermo, Italy

**Keywords:** Geriatric asthma, Airflow obstruction, Lung function tests, Senile lung, Comorbidities

## Abstract

Historically, asthma has been envisioned as a disease of younger ages. This has led to the assumption that respiratory symptoms suggestive of asthma occurring in older ages are to be attributed to conditions other than asthma, mainly COPD. Old observational reports and new epidemiological studies confirm that asthma is as frequent in older as it is in younger populations. Nevertheless, under-recognition, misdiagnosis and under-treatment are still relevant issues. The characterization of asthma in the aged suffers from the fact that there has been very little original research in this field. Indeed, geriatric asthma is often excluded from clinical trials because of age and comorbidities. The current review paper revises the areas that need to be elucidated, and highlights the gaps in the management of this condition. It follows that a multidimensional management is advocated for elderly asthmatics to evaluate the severity and establish the complexity of the disease. We suggest that the term “geriatric” asthma should be preferred to “senile” asthma, which is confined to the age-related changes in the lung, or the more generic “asthma in the elderly”, which is only descriptive of the prevalence in specific age groups.

## Background

The geriatric patient undergoes age-related structural and physiological changes of the lung that strongly influence the features of airway obstruction, which appears more severe and less reversible in response to treatment. Several studies have demonstrated that asthma in older individuals is characterized by a greater reduction in lung function, with lower FEV_1_% predicted as opposed to younger asthmatics. This could contribute to identify a subgroup of severe asthmatics in whom age-related changes are predominant. In addition to this, the acute response to bronchodilators is usually (not always) blunted, and features of reversibility of bronchial obstruction are therefore less prevalent in this age range.

The occurrence of comorbid conditions in geriatric asthmatics is the norm. Physicians are therefore forced to face with the interaction of multiple diseases, and the success of asthma treatment is expected to fail if the comorbidities are not treated appropriately, or at least taken into consideration in the medical decisions. The latter includes several aspects: comorbidities can influence the absorption, distribution, metabolism and excretion of anti-asthmatic drugs. Similarly, the non-respiratory drugs can interact to a variable extent with the respiratory ones, influencing their pharmacokinetics and pharmacodynamics. If these aspects are not properly addressed in clinical practice, the risk of treatment failure and occurrence of systemic and local side effects becomes relevant.

In the current review, we aim at revising the areas that need to be further elucidated from a clinical standpoint, and highlights the major challenges in the management of geriatric asthma.

### Lung functional issues in the diagnosis of geriatric asthma

The persistently higher, and poorly reversible, airway obstruction poses a challenge in the diagnosis of asthma: the risk of misdiagnosis or underdiagnosis is around the corner, and physicians should avoid the risk of following false myths, such as the fact that the lack of reversibility excludes asthma. Findings from the Sa.R.A. Study (Italian acronym for Respiratory Health in the Elderly) showed that only one out of two elderly subjects with carefully ascertained asthma had received a correct diagnosis; conversely, the 20 % of the sample had reported an erroneous diagnosis of COPD [1]. There are reasons why asthma in the elderly loses its “young” characteristics: the lung parenchyma in the elderly undergoes an homogeneous enlargement of airspaces without destruction [2]. The senile lung can be easily distinguished from the emphysematous lung, where the airspace enlargement has an irregular distribution with destruction. This has also been demonstrated by using the lung density by computed tomography [3]. Consequently, the static elastic recoil pressure of the lung diminishes [4], causing reduction in flow rates and vital capacity. The increased compressibility of the airways with age becomes evident at low lung volumes, where the reduction in lung elastic recoil overcomes that of the airways, leading to airflow limitation and airway closure. Obviously, the remodelling changes of the airways with increased thickness of the airway wall oppose the dilating effect. These alterations are not susceptible to the effects of treatment, accounting for the loss of reversibility and the persistence of airway obstruction. The lack of functional reversibility is likely related also to the reduced responsiveness of airway beta_2_-adrenoceptors, which has been demonstrated in healthy elderly subjects [5].

In the evaluation of the functional determinants of asthma in the elderly, the short-term response to a course of oral corticosteroids, or the long-term response to inhaled corticosteroids (ICS) could be a substitute (or a surrogate) of the acute response to short-acting bronchodilators. This is an interesting area of research in the geriatric population. One of the limitations (again, a false myth) is the difficulty in obtaining an optimal lung function assessment by means of spirometry. By no doubt, spirometry is an effort-dependent maneuver, and the collaboration of the patient together with the interaction between the patient and the operator are mandatory. One is therefore led to believe that elderly subjects, by definition, cannot perform a good-quality spirometry. At variance with this misconception, the Sa.R.A. study clearly demonstrated that a good quality test can be obtained in the vast majority of elderly individuals suffering from asthma, provided that the personnel is specifically trained [6]; it was also observed that the performance progressively improves with increasing experience. This emphasizes the opportunity to use reliable surrogate for the forced vital capacity (FVC), such as the forced expiratory volume in six seconds (FEV_6_), to improve the capabilities of lung function assessment in the elderly. Bellia et al. [7] were able to demonstrate, in a large sample of elderly subjects, that FEV_6_ is more easily achievable and more reproducible than FVC; indeed, FEV_6_ was obtained in 82.9 % of spirometries with acceptable start of test, while FVC only in 56.9 %. A good reproducibility (difference between the best two values <5 %) was found in 88.5 % of tests for FEV_6_ and in 86.1 % for FVC, whereas the corresponding figures in obstructed patients were 84.3 % and 75.9 %, respectively.

Beside the issues related to the performance, the critical limitations of the spirometry in the geriatric population are represented by the choice of suitable cut-off diagnostic levels and the lack of appropriate reference values. It is highly recommended that the lower limit of normality is employed to interpret the spirometric results in the geriatric population. There is evidence that the fixed cut-off value of 0.70 for FEV_1/_FVC becomes inaccurate in males aged >40 years and females >50 years [8], implying the risk of an overestimation of the airway obstruction. This has been shown particularly in asymptomatic older individuals with no history of exposure to risk factors and respiratory symptoms, in whom an age-related decline in lung volumes is observed [9].

Since elastic recoil is considered a limiting factor for the maximum decrease in airway caliber during bronchoconstriction [10], the age-associated alterations in lung properties may result in enhanced airway response to spasmogens, which is a cardinal feature of geriatric asthma. Airway hyperresponsiveness (AHR) is not rare amongst the elderly, the reasons being geometrical factors associated with reduced airway caliber, history of smoke exposure and atopy [11]. The evaluation of AHR does not add any value to the diagnostic approach in the geriatric individual from a clinical standpoint; however, the phenomenon of exaggerated airway closure documented in the elderly asthmatics [12], meaning a greater reduction in FVC than in FEV_1_ during a bronchoprovocation challenge, implies that elderly are at higher risk of dramatic episodes of bronchospasms, which can even be letal in some circumstances. Whether this is worth exploring in clinical settings should be decided on specific cases, and research should aim at finding simpler surrogates for the detection of AHR in elderly asthmatics.

Fabbri et al. [13] showed that asthma in the elderly maintains a markedly different pathology compared to COPD, even in the presence of non reversible airway obstruction. In line with this, another Italian study found that despite similar fixed airflow obstruction, elderly patients with asthma have distinct inflammatory pattern in induced sputum and in peripheral blood compared with patients with COPD [14]. These observations raise the need to search for novel biomarkers that might allow to distinguish asthma from COPD in the geriatric ages, and to separate asthma from the asthma-COPD overlap syndrome.

Although the prevalence of atopy is reduced with aging [15], atopy still plays a primary role in the most advanced ages [16]. In other words, despite a decreased prevalence of atopy, the severity of allergic conditions may be increased in older age. For example, an highest risk for asthma hospitalization when atopy and viral infections are combined has been described [17]. Specific interventions aiming at reducing the exposure to environmental stimuli, or specific allergy treatment (e.g. immunotherapy or anti-IgE treatment) are candidate areas for specifically designed studies.

### The challenge of comorbidities in the management of geriatric asthma

The prevalence of chronic degenerative diseases tends to increase with age [18]; an interesting area of research is to explore whether multiple comorbid conditions share a common pathway; for instance, the metabolic syndrome could be pathogenetically linked to asthma through the pro-inflammatory low density lipoproteins, (LDL) as suggested by Scichilone et al. [19] and by Barochia et al. [20]. Small LDL could lead to the amplification of the inflammatory cascade in asthma: larger studies specifically designed to confirm the association between asthma and dyslipidemia in the geriatric asthma are strongly needed. In a cohort of older individuals, Soriano et al. [21] demonstrated an association between asthma and diseases of different organ systems (especially cardiac), with an increased risk of myocardial infarction and angina. Similarly, the association between asthma and atrial fibrillation has been recently shown [22], suggesting that asthma may initiate atrial fibrillation. Moreover, the current use of systemic and inhaled corticosteroids and bronchodilators appeared to be linked to the increased risk of atrial fibrillation in elderly individuals with asthma; on the other hand, medications often used by elderly patients, like ß-adrenergic blockers, might elicit or worsen bronchoconstriction.

In addition to the role of comorbidities in affecting pharmacological treatment, comorbid conditions may impair the ability to use inhalation devices [23]; this is the case for *arthritis* of hands and fingers, which causes hand grip problems, *visual or cognitive impairments* that affect the ability to properly use the inhaled device, and changes in the state of mood (*anxiety and depression*) which can severely reduce the adherence to treatment. In this respect, it is logical to assume that comorbidities and complex treatment regimens can negatively influence the compliance of the asthmatic patient, thus limiting the efficacy of treatment in this age range. It follows that the choice of the proper inhaler device is as important as the choice of the active drug, and should be based on the assessment of coordination between activation and inhalation as well as of the inspiratory flow, together with the evaluation of visual or cognitive impairments. Unfortunately, the progresses in technology in the development of new devices do not seem to have incorporated the needs of the geriatric patients. It is auspicable that specific practical advices and recommendations for the selection of the inhaler device in geriatric asthma would be implemented in clinical daily practice.

## Conclusions

The above-described pitfalls and challenges in geriatric asthma call for a different approach to this pathological condition. It is now accepted that the management of asthma in the elderly should switch from a disease-oriented scheme to a dysfunction-oriented behaviour. In addition to a comprehensive lung function assessment, a multidimensional approach with a multidisciplinary intervention is suggested for elderly asthmatics to evaluate the severity and establish the complexity of the disease. For these reasons, we suggest that the term “geriatric” asthma should be preferred to “senile” asthma, which is confined to the age-related changes in the lung, or the more generic “asthma in the elderly”, which is only descriptive of the prevalence in specific age groups. Geriatric asthma is no longer an enigma to be solved, nor is an “orphan” disease: on the contrary, accumulating evidence confirm that the time has come for a specific (multidimensional) management to be implemented in clinical practice.

### Ethics approval and consent to participate

Not applicable.

## Abbreviations

AHR: Airway hyperresponsiveness
COPD: Chronic obstructive pulmonary disease
FEV1: Forced expiratory volume in the 1st second
FEV6: Forced expiratory volume in six seconds
FVC: Forced vital capacity
ICS: Inhaled corticosteroids
LDL: Low density lipoproteins
Sa.R.A: Salute Respiratoria nell’Anziano, italian for “Respiratory Health in the Elderly”

## References

[1] Bellia V, Battaglia S, Catalano F, Scichilone N, Antonelli Incalzi R, Claudio Imperiale C (2003). Aging and disability affect misdiagnosis of copd in elderly asthmatics: The SARA Study. Chest.

[2] Verbeken EK, Cauberghs M, Mertens I, Clement J, Lauweryns JM, Van de Woestijne KP (1992). The senile lung. Comparison with normal and emphysematous lungs. 1. Structural aspects. Chest.

[3] Bellia M, Benfante A, Menozzi M, Augugliaro G, Scichilone N, Cannizzaro F (2011). Validation of lung densitometry threshold at CT for the distinction between senile lung and emphysema in elderly subjects. Monaldi Arch Chest Dis.

[4] Janssens JP, Pache JC, Nicod LP (1999). Physiological changes in respiratory function associated with ageing. Eur Respir J.

[5] Gupta P, O’Mahony MS (2008). Potential adverse effects of bronchodilators in the treatment of airways obstruction in older people: recommendations for prescribing. Drugs Aging.

[6] Bellia V, Pistelli R, Catalano F, Antonelli-Incalzi R, Grassi V, Melillo G (2000). Quality control of spirometry in the elderly. The SA.R.A. Study. SAlute Respiratoria nell'Anziano: Respiratory Health in the Elderly. Am J Respir Crit Care Med.

[7] Bellia V, Sorino C, Catalano F, Augugliaro G, Scichilone N, Pistelli R (2008). Validation of FEV6 in the elderly: correlates of performance and repeatability. Thorax.

[8] Hankinson JL, Odencrantz JR, Fedan KB (1999). Spirometric reference values from a sample of the general U.S. population. Am J Respir Crit Care Med.

[9] Hardie JA, Buist AS, Vollmer WM, Ellingsen I, Bakke PS, Mørkve O (2002). Risk of over-diagnosis of COPD in asymptomatic elderly never-smokers. Eur Respir J.

[10] Ding DJ, Martin JG, Macklem PT (1987). Effects of lung volume on maximal methacholine-induced bronchoconstriction in normal humans. J Appl Physiol.

[11] Scichilone N, Messina M, Battaglia S, Catalano F, Bellia V (2005). Airway hyperresponsiveness in the elderly: prevalence and clinical implications. Eur Respir J.

[12] Cuttitta G, Cibella F, Bellia V, Grassi V, Cossi S, Bucchieri S (2001). Changes in FVC during methacholine-induced bronchoconstriction in elderly patients with asthma: bronchial hyperresponsiveness and aging. Chest.

[13] Fabbri LM, Romagnoli M, Corbetta L, Casoni G, Busljetic K, Turato G (2003). Differences in airway inflammation in patients with fixed airflow obstruction due to asthma or chronic obstructive pulmonary disease. Am J Respir Crit Care Med.

[14] Di Lorenzo G, Mansueto P, Ditta V, Esposito-Pellitteri M, Lo Bianco C, Leto-Barone MS (2008). Similarity and differences in elderly patients with fixed airflow obstruction by asthma and by chronic obstructive pulmonary disease. Respir Med.

[15] Scichilone N, Callari A, Augugliaro G, Marchese M, Togias A, Bellia V (2011). The impact of age on prevalence of positive skin prick tests and specific IgE tests. Respir Med.

[16] Scichilone N, Augugliaro G, Togias A, Bellia V (2010). Should atopy be assessed in elderly patients with respiratory symptoms suggestive of asthma?. Expert Rev Respir Med.

[17] Heymann PW, Carper HT, Murphy DD, Platts-Mills TA, Patrie J, McLaughlin AP (2004). Viral infections in relation to age, atopy, and season of admission among children hospitalized for wheezing. J Allergy Clin Immunol.

[18] Van den Akker M, Buntinx F, Metsemakers JF, Roos S, Knottnerus JA (1998). Multimorbidity in general practice: prevalence, incidence, and determinants of co-occurring chronic and recurrent diseases. J Clin Epidemiol.

[19] Scichilone N, Rizzo M, Benfante A, Catania R, Giglio RV, Nikolic D (2013). Serum low density lipoprotein subclasses in asthma. Respir Med.

[20] Barochia AV, Kaler M, Cuento RA, Gordon EM, Weir NA, Sampson M (2015). Serum apolipoproteina-I and large high-density lipoprotein particles are positively correlated with FEV1 in atopic asthma. Am J Respir Crit Care Med.

[21] Soriano JB, Visick GT, Muellerova H, Payvandi N, Hansell AL (2005). Patterns of comorbidities in newly diagnosed COPD and asthma in primary care. Chest.

[22] Chan WL, Yang KP, Chao TF, Huang CC, Huang PH, Chen YC (2014). The association of asthma and atrial fibrillation--a nationwide population-based nested case-control study. Int J Cardiol.

[23] Hanania NA, Sharma G, Sharafkhaneh A (2010). COPD in the elderly patient. Semin Respir Crit Care Med.

